# Treatment of stroke patients in the context of the COVID-19 pandemic: Lessons learnt from a major stroke center in Vietnam

**DOI:** 10.7189/jogh.11.03092

**Published:** 2021-08-07

**Authors:** Duy Ton Mai, Hoang Phan, Van Minh Hoang, Tien Dung Nguyen, Ha Quan Phan, Xuan Trung Vuong, Viet Phuong Dao

**Affiliations:** 1 Stroke Center, Bach Mai Hospital, Ha Noi, Vietnam; 2 Menzies Institute for Medical Research, University of Tasmania, Tasmania, Australia; 3 Hanoi University of Public Health, Hanoi, Vietnam

The coronavirus disease 2019 (COVID-19) pandemic is placing enormous strain on the global health care systems. The pandemic has led to a global decline in the number of stroke hospitalizations and interventions (eg, thrombolytic rate reduced by 13%) [1]. The COVID-19 outbreak is continuing to spread around the world, with about 190 million confirmed cases and more than four million deaths across more than 200 countries [2]. For patients with acute stroke regardless of COVID-19 infection, evidence-based care is essential [3]. Country-specific strategies have been undertaken to respond to the pandemic to ensure access to treatment and care for stroke patients [4-6].

The first COVID-19 infection was recorded in Vietnam on January 23, 2020. The country has entered the fourth wave of the COVID-19 pandemic from late-April 2021 with an exponential increase in the number of cases, mostly due to the rapid spread of new coronavirus variants. At the time of writing this paper (June 10, 2021), there had been 6595 cases confirmed in the last 45 days (n = 4888 in two provinces to the east of Hanoi: Bac Ninh and Bac Giang) out of a total of 8165 registered cases across the country since inception [7].

Bach Mai Hospital (BMH) is a major hospital in Hanoi, Vietnam. The stroke center at BMH has the largest catchment of patients across Northern Vietnam [8] with ~ 800 stroke admissions per month pre-COVID. The center provides a wide range of evidence-based stroke care services, such as intravenous thrombolysis, mechanical thrombectomy, and coiling/clipping. During the fourth wave of the pandemic in Vietnam, the number of cases has been exponentially increasing, particular in Ha Noi and other surrounding provinces. To limit the spread of COVID-19, travel restrictions are in place across different cities and provinces in Vietnam. With the fast-changing situation, the risk of having F0 (the infected person), F1 (close contact with F0, or suspected of being infected), and F2 (close contact with F1) presented to our Stroke Center at BMH is inevitable. To avoid the risk of closing the Stroke Center, as was the situation in the 1^st^ wave of the pandemic [8], strategies have been implemented to maintain the best practice stroke care.

In this article, we share our experiences of the Stroke Center of BMH in the treatment of stroke patients in the context of the COVID-19 pandemic in Vietnam that could be useful for similar settings in other countries. Two main strategies have been employed to retain our stroke care services: 1) strengthening telemedicine to provide support for other hospitals that treat stroke; and 2) using a shipping-container Isolation Unit to maintain the treatment for stroke.

**Figure:**
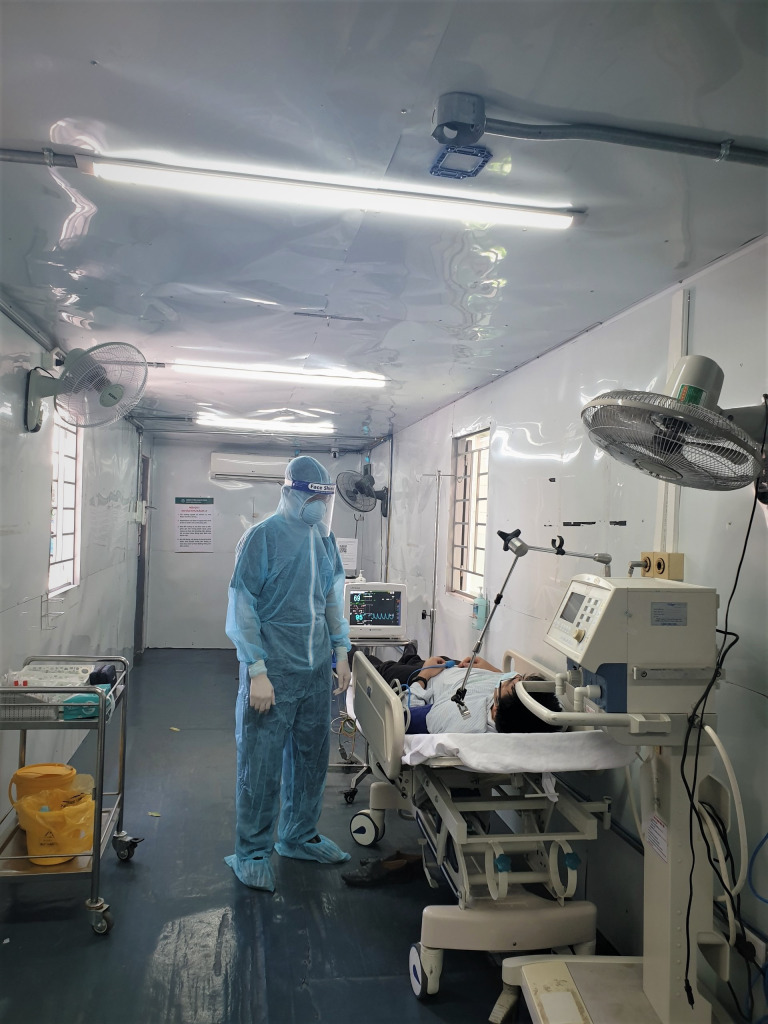
Photo: Treatment of stroke patients in the context of the COVID-19 pandemic at Bach Mai Hospital. Source: http://bachmai.gov.vn/.

Our first approach is to strengthen telemedicine by using virtual consultations that connect ~ 700 stroke doctors from all hospitals across Northern Vietnam to pre-hospital triage patients with suspected stroke. During the 4^th^ wave, only those in need of urgent medical interventions should be admitted to the stroke center. This includes patients with 1) subarachnoid hemorrhages requiring coiling/clipping, 2) intracerebral or intraventricular hemorrhage for which an intervention is indicated. For ischemic stroke (IS), only those with large artery occlusion occurring within the 0-6 hours window and potentially eligible for mechanical thrombectomy are prioritized for admission to a stroke center. The patients must be tested for COVID-19 prior to transfer with results being reported to the telemedicine team during the transfer. Patients with an IS other than large artery occlusion are to be treated with intravenous thrombolysis, if eligible, under the guidance of virtual consultations with the telemedicine team at local hospitals.

**Figure 1:**
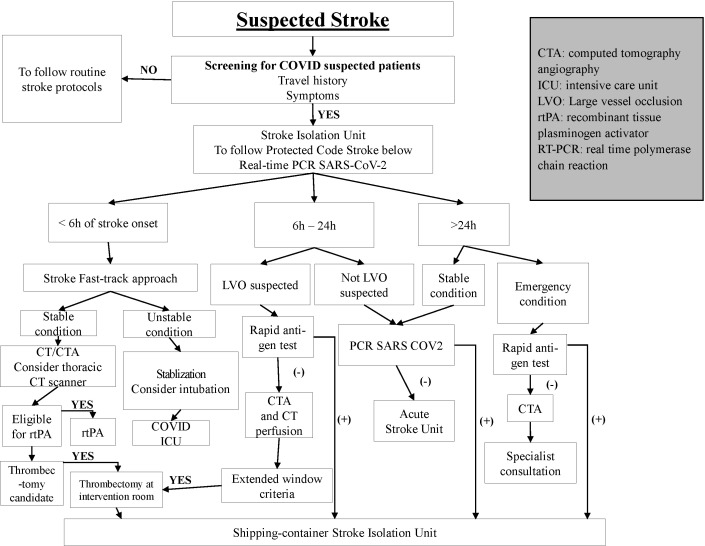
Flow-chart for triage stroke patients.

We have developed a local protocol for triaging stroke patients during the pandemic (**Figure 1**). All the patients transferred from other hospitals or facilities should be presented to our Stroke Triage Unit, which is located outside the stroke center. Those who are F0, F1, or F2 are taken to the Stroke Isolation Unit (SIU) – a newly established unit for further evaluation and treatment. The patients then have a brain imaging at the Department of Diagnostic Imaging and Intervention (DDII) at BMH using special arrangements for intra-hospital transfer (eg, via a special route of movement) and a designated intervention room (with restricted access) within the DDII for suspected COVID patients. If an intravenous thrombolysis is indicated, the patient is to be treated at the SIU. For those who are eligible and indicated, endovascular thrombectomy is performed within the DDII, with separate intervention rooms for COVID suspected patients and the remaining patients. All staff in the SIU are equipped with the highest level of transmission-based precautions, eg, personal protective equipment as well as walkie-talkie system for communications.

**Figure 2:**
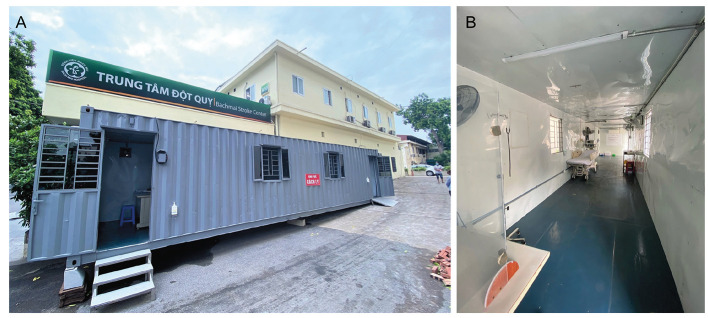
Shipping-container Stroke Isolation Unit at Bach Mai Hospital. **Panel A**. Front view. **Panel B**. Inside view.

Our shipping-container SIU (**Figure 2**) was established on the April 28, 2021 to minimize the risk of a hospital cluster outbreak of COVID-19. The unit is equipped with air conditioning, oxygen, and compressed air systems for mechanical ventilation resuscitation. The cost for renting the shipping-container is about 175 USD/month ( ~ 4 000 000 Vietnam Dong). The mobile SIU was set up within 2 days in an isolated area within our stroke center to ensure cross-infection control of COVID-19 for frontline health care workers and patients.

Our local statistics show that the proportions of patients receiving reperfusion (IS) and clipping or coiling (hemorrhage stroke) were higher in May 2021 compared to the figures of earlier months (**Table 1**). This is likely due to the number of stroke admissions decreased significantly at 1 month following the 4^th^ wave occurrence. The potential reasons for the decline in stroke hospitalizations are a) patient fear of contracting COVID-19 [1], and b) physical distancing measures that may prevent people suffering stroke from timely seeking medical care [1], and c) only those in need of urgent medical interventions were hospitalized. The absolute number of interventions performed in May, although being smaller than that of the period preceding the pandemic wave, reflected our ability to maintain the best practice stroke care.

**Table 1:** Stroke admissions and intervention to the Stroke Center at Bach Mai Hospital in 2021

	Hospital admissions	Stroke center admission		Reperfusion intervention performed (thrombolysis/thrombectomy)		Neurosurgery performed (clipping/coiling)	
	n	n	%	n	%	n	%
January	13 015	817	6.28	45	5.5	47	5.75
February	5569	569	10.22	38	6.67	34	5.97
March	12 211	790	6.47	58	7.34	59	7.46
April	12 270	825	6.72	68	8.24	51	6.18
May	5023	334	6.65	39	11.67	25	7.48

Our shipping-container SIU at BMH is the first purpose-built unit for isolation of stroke patients with suspected COVID-19 to be used in a hospital setting in Vietnam. The findings provide evidence that our strategies, including the use of telemedicine, triage protocols, and the shipping-container SIU have brought benefits in maintain best practice care for patients with acute stroke at our center during the era of COVID pandemic. Our experiences could be useful for similar settings in other countries around the world.
